# Genomic insights into the diversity, virulence, and antimicrobial resistance of group B *Streptococcus* clinical isolates from Saudi Arabia

**DOI:** 10.3389/fcimb.2024.1377993

**Published:** 2024-04-22

**Authors:** Maha Alzayer, Manal M. Alkhulaifi, Ahmed Alyami, Mohammed Aldosary, Abdulaziz Alageel, Ghada Garaween, Atef Shibl, Arif M. Al-Hamad, Michel Doumith

**Affiliations:** ^1^ Department of Botany and Microbiology, College of Science, King Saud University, Riyadh, Saudi Arabia; ^2^ Department of Microbiology and Immunology, College of Medicine, Alfaisal University, Riyadh, Saudi Arabia; ^3^ Pathology and Clinical Laboratory, Medicine Administration, King Fahad Medical City, Riyadh, Saudi Arabia; ^4^ Division of Clinical Microbiology, Pathology and Laboratory Medicine, Qatif Central Hospital, Qatif, Saudi Arabia; ^5^ Infectious Diseases Research Department, King Abdullah International Medical Research Center, Riyadh, Saudi Arabia

**Keywords:** group B streptococcus, antimicrobial resistance, whole-genome sequencing, MLST, CC17, ST1212, HvgA, Saudi Arabia

## Abstract

**Introduction:**

Detailed assessment of the population structure of group B *Streptococcus* (GBS) among adults is still lacking in Saudi Arabia. Here we characterized a representative collection of isolates from colonized and infected adults.

**Methods:**

GBS isolates (n=89) were sequenced by Illumina and screened for virulence and antimicrobial resistance determinants. Genetic diversity was assessed by single nucleotide polymorphisms and core-genome MLST analyses.

**Results:**

Genome sequences revealed 28 sequence types (STs) and nine distinct serotypes, including uncommon serotypes VII and VIII. Majority of these STs (n=76) belonged to the human-associated clonal complexes (CCs) CC1 (33.71%), CC19 (25.84%), CC17 (11.24%), CC10/CC12 (7.87%), and CC452 (6.74%). Major CCs exhibited intra-lineage serotype diversity, except for the hypervirulent CC17, which exclusively expressed serotype III. Virulence profiling revealed that nearly all isolates (94.38%) carried at least one of the four alpha family protein genes (i.e., *alphaC*, *alp1*, *alp2/3*, and *rib*), and 92.13% expressed one of the two serine-rich repeat surface proteins Srr1 or Srr2. In addition, most isolates harbored the pilus island (PI)-2a alone (15.73%) or in combination with PI-1 (62.92%), and those carrying PI-2b alone (10.11%) belonged to CC17. Phylogenetic analysis grouped the sequenced isolates according to CCs and further subdivided them along with their serotypes. Overall, isolates across all CC1 phylogenetic clusters expressed Srr1 and carried the PI-1 and PI-2a loci, but differed in genes encoding the alpha-like proteins. CC19 clusters were dominated by the III/*rib*/*srr1*/PI-1+PI-2a (43.48%, 10/23) and V/*alp1*/*srr1*/PI-1+PI-2a (34.78%, 8/23) lineages, whereas most CC17 isolates (90%, 9/10) had the same III/*rib*/*srr2*/P1-2b genetic background. Interestingly, genes encoding the CC17-specific adhesins HvgA and Srr2 were detected in phylogenetically distant isolates belonging to ST1212, suggesting that other highly virulent strains might be circulating within the species. Resistance to macrolides and/or lincosamides across all major CCs (n=48) was associated with the acquisition of *erm(B)* (62.5%, 30/48), *erm(A)* (27.1%, 13/48), *lsa(C)* (8.3%, 4/48), and *mef(A)* (2.1%, 1/48) genes, whereas resistance to tetracycline was mainly mediated by presence of *tet(M)* (64.18%, 43/67) and *tet(O)* (20.9%, 14/67) alone or in combination (13.43%, 9/67).

**Discussion:**

These findings underscore the necessity for more rigorous characterization of GBS isolates causing infections.

## 1 Introduction

Group B *Streptococcus* (GBS) is primarily known as a commensal pathogen that resides asymptomatically in the gastrointestinal and genitourinary tracts of over 30% of healthy adults (Raabe and Shane, 2019; Chen et al., 2023). However, the species can sometimes cause serious illnesses, leading to death among susceptible hosts, including neonates, pregnant women, and non-pregnant adults, especially the elderly and those with underlying health conditions (Furfaro et al., 2018). The incidence of GBS infections has been increasingly reported over the last two decades, with an estimated rate ranging from 3.6 to 7.3 cases per 100,000 inhabitants and a case-fatality rate of over 15% (Skoff et al., 2009). Penicillin remains the first-line drug for the treatment of GBS infections. However, reduced susceptibility to penicillin associated with mutations in penicillin-binding proteins (PBPs) has been reported (Kimura et al., 2008; McGee et al., 2021). Erythromycin, clindamycin, and levofloxacin are recommended as alternative agents for patients with β-lactam allergies, but non-susceptibility to these antibiotics has also been documented globally, thus limiting their use in the treatment of GBS infections (Castor et al., 2008; American College of Obstetricians and Gynecologists, 2020). Resistance to macrolides and lincosamides in GBS is mainly associated with the acquisition of methyltransferases encoded by the *erm* genes or efflux pumps encoded by the *mef*, *msr*, or *lsa* genes, whereas resistance to fluoroquinolones is caused by alterations in the targets GyrA and ParC (Leclercq, 2002; Metcalf et al., 2017; Hayes et al., 2020).

The transition from commensal to pathogenic GBS involves an array of multifunctional virulence factors. Among these, the capsular polysaccharide (CPS), which is the most studied factor, defines GBS serotypes and plays a critical role in immune evasion (Shabayek and Spellerberg, 2018). To date, ten distinct serotypes (i.e., Ia, Ib, and II to IX) have been identified in the species, of which, serotypes Ia, III, and V are the most frequent in adult carriage and disease (Bianchi-Jassir et al., 2020). GBS also possesses a pilus-like structure that mediates GBS colonization and adhesion to host cells. Two different pilus islands (PI), namely PI-1 and PI-2, with the latter divided into PI-2a and PI-2b variants, have been identified. All GBS strains harbor one or two PIs, with PI-2a and PI-2b being mutually exclusive (Springman et al., 2014). Other surface proteins, including the alpha-like protein family (Alpha C, Alp1, Alp2/3, and Rib), serine-rich repeat protein (Srr), C5a peptidase (ScpB), laminin-binding surface protein (Lmb), hypervirulent adhesin (HvgA), and fibrinogen-binding protein (Fbs) have also been reported to contribute to host cell adherence and invasion (Tazi et al., 2010; Furfaro et al., 2018; Shabayek and Spellerberg, 2018).The population structure of GBS has been studied using various techniques including multilocus sequence typing (MLST). Six major clonal complexes (CCs), namely, CC1, CC10/CC12, CC17, CC19, CC23, and CC26, have been largely associated with asymptomatic colonization and GBS infections (Skov Sørensen et al., 2010; Da Cunha et al., 2014). Of these, CC17 is strongly linked to CPS III and has been globally associated with invasive diseases and meningitis in infants, while CC1, CC10/CC12, CC19, and CC23 are frequent colonizers and can present with multiple serotypes (Da Cunha et al., 2014).

In recent years, genome-based studies on GBS isolates have provided a more comprehensive understanding of the epidemiology of this species (Kapatai et al., 2017; Meehan et al., 2021). Although whole-genome sequencing (WGS) has been widely used for the characterization of GBS isolates, no published genome sequences from Saudi Arabia are available. Here, we used WGS to characterize the population structure of colonizing and infecting GBS isolates recovered from pregnant women and non-pregnant adults. In particular, this study aimed to comprehensively analyze the distribution of genetic features associated with virulence and resistance to antibiotics and their relationship with GBS genomic lineages that are currently circulating in the kingdom. Ultimately, the generated data will enrich public databases by contributing with WGS input from Saudi isolates.

## 2 Materials and methods

### 2.1 GBS isolates

Eighty-nine GBS isolates were selected to represent the diversity of a recently published collection of 204 clinical isolates from infected (n = 95) and colonized adults (n = 109) aged between 15 and 95 years old (Alzayer et al., 2023). Published isolates comprised six different serotypes, including serotypes III (25%), V (25%), II (16.8%), Ia (13.24%), VI (9.31), Ib (8.82%) and five (2.45%) that were none typeable by standard PCR. These clinical isolates were recovered from urine (n = 108), rectovaginal swabs (n = 73), wound swabs (n = 12), soft tissues (n = 5), blood (n = 5), and bones (n = 1) over the period of February to September 2022 from three hospital settings located in the central region (i.e., C1 and C2), and eastern region (i.e., E1) of Saudi Arabia. Initial susceptibility testing by disk diffusion assays identified resistance to tetracycline (76.47%), erythromycin (36.76%), clindamycin (25.49%), and levofloxacin (6.37%). Sequenced isolates in this study (n = 89) covered serotypes III (26.97%, n = 24), V (21.35%, n = 19), II (13.48%, n = 12), Ib (12.36%, n = 11), VI (11.24%, n = 10), Ia (8.99%, n = 8), and all those that were non-typeable in the previously published collection (5.62%, n = 5) (Alzayer et al., 2023). In addition, they were chosen to represent all the resistance profiles observed in isolates associated with colonization (n = 42), non-invasive (n = 42), and invasive infections (n = 5) as detailed in the **Supplementary Table 1**. Of note, the selected isolates included two pairs from the same patients retrieved from different specimen types (**Supplementary Table 1**).

### 2.2 Whole genome sequencing and bioinformatics

Genomic DNA of GBS isolates was extracted using the silica column of the QIAamp DNA Mini Kit (QIAGEN, Hilden, Germany) as previously described (Alzayer et al., 2023). Good-quality DNA was then prepared for sequencing on the NovaSeq6000 v1.5 Illumina platform (Illumina, San Diego, CA, USA) using the 2 x150 bp paired-end sequencing protocol. Raw read sequences were checked using FastQC v0.11.9 (https://www.bioinformatics.babraham.ac.uk/projects/fastqc/) and *de novo* assembled using SPAdes software (Version 3.14.1) (Bankevich et al., 2012). The quality of the assembled contigs was evaluated using QUAST v5.0.2 (http://bioinf.spbau.ru/en/quast) and all identified contigs shorter than 500 bp were filtered prior to further analysis (Gurevich et al., 2013). The bacterial species were confirmed using Kraken2 v2.0.8 (https://github.com/DerrickWood/krake) (Wood et al., 2019). Capsular genotyping was performed by mapping the reads against previously published GBS capsular locus sequences (https://github.com/swainechen/GBS-SBG) (Tiruvayipati et al., 2021). Sequence types (STs) were predicted in silico from the assembled contigs using the MLST software (Version 2.17.6) (https://github.com/tseemann/mlst) and the *S. agalactiae* PubMLST reference database (https://pubmlst.org/sagalactiae/). Novel allele sequences and allelic combinations were submitted to the PubMLST database (https://pubmlst.org/). Acquired antimicrobial resistance genes were extracted from the assembled contigs using ABRicate v0.9.8 (https://github.com/tseemann/abricate) together with Resfinder and NCBI databases and then confirmed by mapping using Gene-finder software (https://github.com/phe-bioinformatics/gene_finder). Alterations in GyrA and ParC and the presence of genes encoding pilus island loci (PI-1, PI-1b, PI-2a, and PI-2b variants), surface proteins (Alpha C, Alp1, Alp2/3, and Rib), serine-rich repeat protein (Srr), and the hypervirulent GBS adhesin (HvgA) (https://github.com/BenJamesMetcalf) and those associated with GBS virulence (VFDB: http://www.mgc.ac.cn/VFs/) were also obtained via ABRicate or by mapping using Gene-finder (Liu et al., 2019). Only genes sharing > 95% identity with the reference sequences have been reported as present (McGee et al., 2021). The presence of the integrative conjugative element ICESag37 in the sequenced genome was determined by checking the depth of coverage of the reads mapped across the full published sequence of this element (ACC: OP508056). The relatedness among the sequenced genomes was assessed using a single nucleotide polymorphism (SNPs)-based approach by mapping reads against the sequence of the publicly available strain COH1 (CP129875.1) with snippy (https://github.com/tseemann/snippy). Filtered SNPs were concatenated and used to construct a phylogenetic tree using IQ-Tree v1.6.9 (Nguyen et al., 2015). The phylogenetic tree was visualized and annotated using the Interactive Tree of Life (iTOL v6.5; https://itol.embl.de/#) (Letunic and Bork, 2007). Relationships between isolates were also determined by core genome cg-MLSTs, which were obtained and visualized using the built-in solutions in EnteroBase (https://enterobase.warwick.ac.uk/species/index/streptococcus). GrapeTree, a minimum-spanning tree, was constructed via EnteroBase GrapeTree using MSTree v2 (Zhou et al., 2018). Differences between cg-MLST profiles were calculated using cgmlst-dist (https://github.com/tseemann/cgmlst-dists). Raw reads were deposited in the European Nucleotide Archive (ENA) (https://www.ebi.ac.uk/ena) under the project accession number PRJEB70279.

## 3 Results

### 3.1 Serotyping

**Figure 1:**
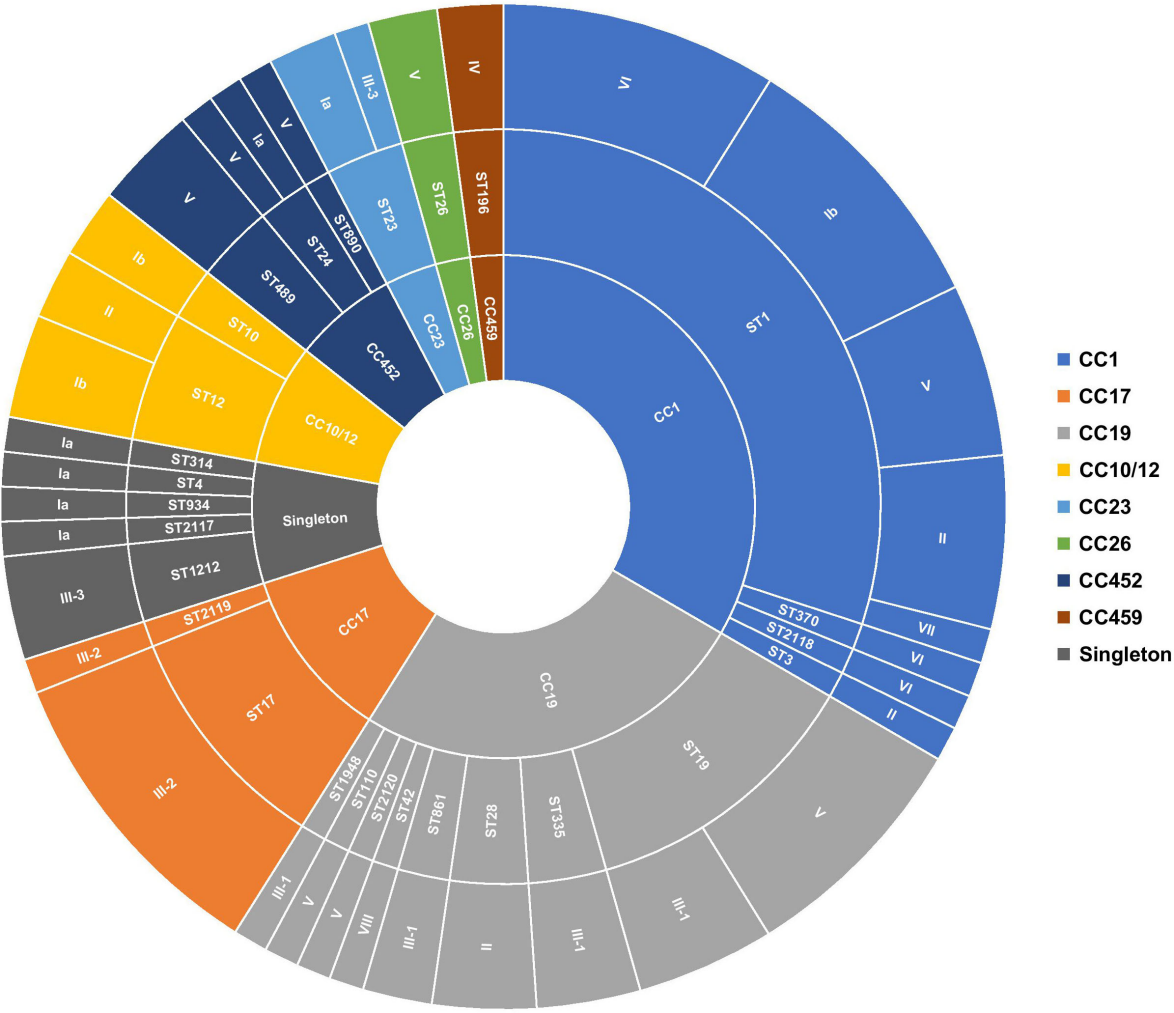
Distribution of serotypes and genetic lineages among GBS isolates. Segments were scaled according to the number of isolates belonging to each genotype. The inner circle represents the CCs, the middle circle represents the STs in relation to each CC, and the outer circle represents the capsular serotypes in relation to each ST.

Nearly all isolates (92.13%, 82/89) previously assigned by PCR to serotypes III (n = 24/82), V (n = 19/82), Ib (n = 11/82), II (n = 11/82), VI (n = 10/82), and Ia (n = 7/82) were identically inferred from genome sequences using the GBS-SBG reference database (Tiruvayipati et al., 2021). Only two isolates assigned to serotypes Ia and II by standard PCR were predicted in silico as serotypes IV and VII, respectively. In addition, the five non-typeable isolates included in the study were inferred as serotypes Ib (n = 2), V (n = 2), and VIII (n = 1) (Alzayer et al., 2023). Sequence analysis further sub-divided serotype III into subtypes III-1 (41.67%, n = 10/24), III-2 (41.67%, n = 10/24), and III-3 (16.67%, n = 4/24) (**Figure 1**, **Supplementary Table 1**).

### 3.2 Genetic lineage distribution

In total, 28 unique sequence types (STs), including four novel STs (i.e., ST2117, ST2118, ST2119, and ST2120), were identified among the sequenced isolates, suggesting high genetic diversity. ST1, the most prevalent sequence type, accounted for 29.21% (n = 26/89) of isolates, followed to a lesser extent by ST19 (12.36%, n = 11/89), ST17 (10.11%, n = 9/89), and ST12 (5.62%, n = 5/89). Of the remaining, ST23, ST28, ST335, ST498, and ST1212 were each represented by three isolates (3.37%, n = 3/89), while 19 STs had at most two representatives each (**Figure 1**; **Supplementary Table 1**). The majority of detected STs (85.39%, n = 76/89) clustered into the major CCs, CC1 (33.71%, n = 30/89), CC10/CC12 (7.87%, n = 7/89), CC17 (11.24%, n = 10/89), CC19 (25.84%, n = 23/89), and CC452 (6.74%, n = 6/89) (**Figure 1**; **Supplementary Table 1**). Furthermore, each detected ST belonged to a single serotype, except for ST1, ST12, ST19, ST23, and ST24, which exhibited up to five different serotypes (**Figure 1**). This was particularly prominent in the ST1 isolates expressing serotypes Ib, II, V, VI, and VII. Overall, there was no clear association between CCs and GBS carriage or disease (**Supplementary Figure 1**).

### 3.3 Molecular mechanisms of antimicrobial resistance

Phenotypic susceptibility testing of sequenced isolates revealed resistance to tetracycline (75.28%, n = 67/89), erythromycin (49.44%, n = 44/89), clindamycin (38.20%, n = 34/89), levofloxacin (12.36%, n = 11/89), and gentamicin (4.49%, n = 4/89) (**Supplementary Table 1**). Erythromycin-resistant isolates were constitutive (33.71%, n = 30/89) or inducible (14.61%, n = 13/89) Macrolide-Lincosamide-Streptogramin B (MLS_B_) phenotypes, and only one isolate exhibited the M phenotype. Four isolates were resistant to clindamycin (4.49%, n = 4/89) and showed the L phenotype (**Table 1**; **Supplementary Table 1**).

**Table 1:** Distribution of antimicrobial resistance profiles among sequenced GBS isolates (n = 89).

Genetic Lineage	Serotype (n)	Macrolide resistance phenotype (n)	Macrolide resistance genotype (n)	Tetracycline resistance genotype (n)	Other antimicrobial resistance genotypes (n)
CC1 (n = 30)					
ST1	Ib (8)	cMLS_B_ (8)	*erm(B)* (8)	*tet(M)* (8)	
	II (5)	iMLS_B_ (2)	*erm(A)* (2)		
				*tet(M)* (3)	
	V (4)	iMLS_B_ (1)	*erm(A)* (1)	*tet(M)* (1)	
				*tet(M)* (3)	
	VI (8)				ParC (S79A) (1)
	VII (1)			*tet(M)* (1)	
ST2	V (1)			*tet(M)* (1)	
ST3	II (1)	iMLS_B_ (1)	*erm(A)* (1)	*tet(M)* (1)	
ST370	VI (1)			*tet(O)* (1)	
ST2118	VI (1)				
CC10/CC12 (n = 7)					
ST10	Ib (2)	cMLS_B_ (2)	*erm(B)* (2)		GyrA (S81L), ParC (S79F) (2)
ST12	Ib (3)	cMLS_B_ (3)	*erm(B)* (3)	*tet(M), tet(O)* (1)	*ant(6)-Ia, aph(3’)-III, aadE* (1)
				*tet(O)* (2)	*ant(6)-Ia, aph(3’)-III, aadE* (2)
	II (2)				ParC (S79F) (1)
CC17 (n = 10)					
ST17	III-2 (7)	cMLS_B_ (7)	*erm(B)* (7)	*tet(M), tet(O)* (4)	*ant (6)-Ia; aph (3’)-III, aadE* (4)
				*tet(O)* (3)	*ant (6)-Ia; aph (3’)-III, aadE* (3)
	III-2 (2)			*tet(M)* (1)	

ST2119	III-2 (1)	cMLS_B_ (1)	*erm(B)* (1)	*tet(M), tet(O)* (1)	*ant (6)-Ia, aph(3’)-III, aadE* (1)
CC19 (n = 23)					
ST19	III-1 (4)	iMLS_B_ (1)	*erm(A), mef(A)/msr(D)* (1)	*tet(M)* (1)	*cat*, GyrA (S81L), ParC (S79F) (1)
		cMLS_B_ (1)	*erm(B), lsa(C)* (1)	*tet(O)* (1)	
		L (1)	*lsa(C)*		
				*tet(O), tet(L)* (1)	*aac(6’)-aph(2’’), ant(6)-Ia, cat*, GyrA (S81L), ParC (S79Y) (1)
	V (7)	cMLS_B_ (2)	*erm(B)* (2)	*tet(M), tet(O)* (2)	
		iMLS_B_ (5)	*erm(A)* (3)	*tet(M)* (3)	*aac(6’)-aph(2’’)*, GyrA (S81L), ParC (S79F) (3)
			*erm(A)* (1)	*tet(M)* (1)	GyrA (S81L), ParC (S79F) (1)
			*erm(A), mef(A)/msr(D)* (1)	*tet(M)* (1)	*cat*, GyrA (S81L), ParC (S79F) (1)
ST28	II (3)	cMLS_B_ (3)	*erm(B)* (3)	*tet(M), tet(O)* (1)	
				*tet(O)* (2)	
ST42	VIII (1)				
ST110	V (1)	cMLS_B_ (1)	*erm(B)* (1)	*tet(O)* (1)	
ST335	III-1 (3)	iMLS_B_ (3)	*erm(A)* (3)	*tet(M)* (3)	
ST861	III-1 (2)	L (2)	*lsa(C)* (2)	*tet(O)* (2)	
ST1948	III-1 (1)	L (1)	*lsa(C)* (1)	*tet(O)* (1)	*aph(3’)-III* (1)
ST2120	V (1)			*tet(M)* (1)	GyrA (S81L), ParC (S79F) (1)
CC23 (n = 3)					
ST23	Ia (1)	M (1)	*mef(A)/msr(D)* (1)	*tet(M)* (1)	
	Ia (1)			*tet(M)* (1)	*aadE* (1)
	III-3 (1)				
CC26 (n = 2)					
ST26	V (2)			*tet(M)* (1)	

CC452 (n = 6)					
ST24	Ia (1)			*tet(M)* (1)	
	V (1)			*tet(M)* (1)	
ST498	V (3)			*tet(M)* (3)	
ST890	V (1)	cMLS_B_ (1)	*erm(B)* (1)	*tet(M*) (1)	*ant (6)-Ia, aph(3’)-III* (1)
CC459 (n = 1)					
ST196	IV (1)	cMLS_B_ (1)	*erm(B)* (1)	*tet(O)* (1)	
singleton (n = 7)					
ST4	Ia (1)				
ST314	Ia (1)			*tet(M)* (1)	
ST934	Ia (1)				
ST1212	III-3 (3)			*tet(M)* (1)	
ST2117	Ia (1)			*tet(M)* (1)	

MLS_B_, resistance to macrolides, lincosamides, and streptogramin B with the prefix letter referring to the constitutive (cMLS_B_) or inducible (iMLS_B_) expression phenotype; M, resistance to macrolides; L, resistance to lincosamides; CCs, clonal complexes; STs, MLST sequence types.

Genome sequence analysis showed that all constitutively resistant isolates (n = 30) harbored the *erm(B)* gene and those expressing the inducible phenotype (n = 13) carried *erm(A)*. Isolates with the M (n = 1) and L (n = 4) phenotypes carried the *mef(A)* or *lsa(C)* genes, respectively. Otherwise, *mef(A)* and *erm(A)* were co-detected in two isolates, and *lsa(C)* was detected in one *erm(B)*-positive isolate (**Table 1**). Resistance to levofloxacin was linked to alterations in both GyrA (Ser81Leu) and ParC (Ser79Phe or Ser79Tyr) in 10 isolates and to Ser79Tyr alteration in only ParC in a single isolate. One isolate belonging to serotype VI carried a Ser79Ala alteration in ParC but remained phenotypically susceptible to levofloxacin. Among the isolates showing resistance to tetracycline (n = 67), 43 (64.18%, n = 43/67) and 14 (20.9%, n = 14/67) harbored *tet(M*) and *tet(O*), respectively, while 10 isolates (14.93%, n = 10/67) carried *tet(O)* with either *tet(M)* (n = 9) or *tet(L*) (n =1). On the other hand, all isolates exhibiting high levels of resistance to gentamicin (n = 4) carried the *aac(6’)-aph(2’’)* gene. Genome screening also detected the presence of the chloramphenicol resistance gene *cat* in three isolates and the aminoglycoside resistance genes *aadE*, *ant (6’)-Ia*, and/or *aph(III’)-Ia* in 15 isolates belonging to CC17/III-2 (n = 8), CC10/CC12/Ib (n = 3), CC19/III-1 (n = 2), CC23/Ia (n = 1), and CC452/V (n = 1) (**Table 1**; **Supplementary Table 1**). In most cases (73.33%, n = 11/15), the three aminoglycosides resistant genes were co-located with *erm(B)* and *tet(O)* in isolates belonging to CC17 (n = 10) and CC10/CC12 (n = 1). In GBS, these five resistance determinants were previously co-located within the integrative conjugative element, ICESag37 (Zhou et al., 2017). Mapping of sequencing reads against the published ICESag37 sequences showed that all isolates carrying these five resistance determinants had the same integrative conjugative element. Overall, all sequenced isolates were susceptible to penicillin, and none had alterations in the binding of bacterial PBPs to β-lactams. Otherwise, 17.98% (n = 16/89) of the isolates did not carry any of the acquired resistance determinants (**Table 1**).

### 3.4 Distribution of pilus island types

At least one PI locus was detected in each sequenced isolate. Most genomes (n = 70) harbored PI-2a alone (15.73%, n = 14/89) or in combination with PI-1 (62.92%, n = 56/89), whereas 18 isolates carried PI-2b alone (10.11%, n = 9/89) or in combination with PI-1 (10.11%, n = 9/89), and one isolate harbored PI-1 alone (1.12%, n = 1/89) (**Table 2**; **Supplementary Table 1**). Notably, sequence analysis showed that nearly a quarter (26.87%, n = 18/67) of PI-1 encoded the recently described PI-1b variant, which was missed during the initial screening using the previously described PCR assay (**Table 2**; **Supplementary Table 1**) (Martins et al., 2010). Overall, clear associations were observed between specific CCs and PIs (**Supplementary Figure 2**). Almost all isolates belonging to CC17 (n = 9/10) carried exclusively PI-2b, while the remaining isolate had PI-1 and PI-2b. Otherwise, majority of isolates belonging to CC1 (96.67%, n = 29/30), CC19 (82.61%, n = 19/23), and CC10/CC12 (85.71%, n = 6/7) carried PI-1 and PI-2a, while all isolates belonging to CC26 (n = 2) and CC452 (n = 6) carried PI-2a alone (**Table 2**; **Supplementary Figure 2**). Moreover, there were clear associations between the PI variants and certain capsular serotypes. Indeed, all serotype VI (n=10) and serotype III-1 isolates (n = 10) and the majority of isolates with serotypes Ib (92.31%, n = 12/13) and II (81.82%, n = 9/11) had PI-1 and PI-2a (**Table 2**; **Supplementary Table 1**). Majority of serotype III-2 (90%, n = 9/10) isolates had only PI-2b, and those belonging to serotype III-3 (75%, n = 3/4) carried both PI-1 and PI-2b (**Table 2**; **Supplementary Table 1**).

**Table 2:** Distribution of pili, surface protein, and virulence factor profiles among GBS serotypes and clonal complexes.

Genetic Lineage	Serotype (n)	Surface proteins					Virulence Factors					
		*alp* gene	*srr* gene	PI-I variant (n)	PI-II variant (n)	*hvgA*	*fbs*	*lmb*	*hylB*	*scpB*	*cfa/cfb*	*cyl* operon*
CC1 (n = 30)												
ST1	Ib (8)	*alp2/3*	*srr1*	PI-1	PI-2a	–	–	+	+	+	+	+
	II (5)	*alp2/3*	*srr1*	PI-1	PI-2a	–	–	+	+	+	+	+
	V (4)	*alp2/3*	*srr1*	PI-1	PI-2a	–	–	+	+	–	+	+
	VI (8)	*alphaC*	*srr1*	PI-1b	PI-2a	–	–	+	+	+	+	+
	VII (1)	*alp2/3*	*srr1*	PI-1	PI-2b	–	–	+	+	+	+	+
ST2	V (1)	*rib*	*srr1*	PI-1	PI-2a	–	–	+	+	–	+	+
ST3	II (1)	*alphaC*	*srr1*	PI-1	PI-2a	–	–	+	+	+	+	+
ST370	VI (1)	*rib*	*srr1*	PI-1b	PI-2a	–	–	+	+	+	+	+
ST2118	VI (1)	*alphaC*	*srr1*	PI-1b	PI-2a	–	–	+	+	+	+	+
CC10/CC12 (n = 7)												
ST10	Ib (2)	*alphaC*	*srr1*	PI-1b	PI-2a	–	–	+	+	+	+	+
ST12	Ib (2)	*alphaC*	–	PI-1	PI-2a	–	–	+	+	+	+	+
	Ib (1)	*alphaC*	–	–	PI-2a	–	–	+	+	+	+	+
	II (2)	*alp1*	–	PI-1b	PI-2a	–	–	+	+	+	+	+
		*alphaC*	*srr1*									
CC17 (n = 10)												
ST17	III-2 (8)	*rib*	*srr2*	PI-1 (1)	PI-2b	+	*fbsB*	+	+	+	+	+
	III-2 (1)	*rib*	*srr2*	–	PI-2b	+	*fbsB*	–	+	–	+	+
ST2119	III-2 (1)	*rib*	*srr2*	–	PI-2b	+	*fbsB*	+	+	+	+	+
CC19 (n = 23)												
ST19	III-1 (4)	*rib*	*srr1*	PI-1	PI-2a	–	–	+	–	+	+	+
	V (7)	*alp1*	*srr1*	PI-1	PI-2a	–	–	+	+	+	+	+
ST28	II (3)	*rib*	–	PI-1 (1)	PI-2a (3)	–	–	+	+	+	+	+
ST42	VIII (1)	*alp1*	*srr1*	PI-1b	PI-2b	–	–	+	+	+	+	+
ST110	V (1)	*rib*	*srr1*	–	PI-2a	–	–	+	+	+	+	+
ST335	III-1 (3)	*rib*	*srr1*	PI-1	PI-2a	–	–	+	+	+	+	+
ST861	III-1 (2)	*rib*	*srr1*	PI-1	PI-2a	–	–	+	–	+	+	+
ST1948	III-1 (1)	*rib*	*srr1*	PI-1	PI-2a	–	–	+	–	+	+	+
ST2120	V (1)	*alp1*	*srr1*	PI-1	PI-2a	–	–	+	+	+	+	+
CC23 (n = 3)												
ST23	Ia (2)	*alp1*	*srr1*	–	PI-2a	–	*fbsB*	+	+	+	+	+
	III-3 (1)	*alp2/3*	*srr1*	PI-1	PI-2a	–	*fbsB*	+	+	+	+	+
CC26 (n = 2)												
ST26	V (2)	–	*srr1*	–	PI-2a	–	–	+	+	+	+	+
CC452 (n = 6)												
ST24	Ia (1)	*alphaC*	*srr1*	–	PI-2a	–	*fbsB*	+	+	+	+	+
	V (1)	*alphaC*	*srr1*	–	PI-2a	–	*fbsB*	+	+	+	+	+
ST498	V (3)	*alphaC*	*srr1*	–	PI-2a	–	*fbsB*	+	+	+	+	+
ST890	V (1)	*alphaC*	*srr1*	–	PI-2a	–	*fbsB*	+	+	+	+	+
CC459 (n = 1)												
ST196	IV (1)	*alp1*	*srr1*	PI-1	PI-2a	–	*fbsA*	+	+	+	+	+
Singleton												
ST4	Ia (1)	*alp1*	*srr1*	PI-1b	PI-2b	–	–	+	+	+	+	+
ST314	Ia (1)	*alp2/3*	*srr1*	PI-1	–	–	–	–	+	–	+	+
ST934	Ia (1)	*alp1*	*srr1*	PI-1b	PI-2b	+	–	+	+	+	+	+
ST1212	III-3 (3)	–	*srr2*	PI-1	PI-2b	+	–	+	+	+	+	+
ST2117	Ia (1)	*alp1*	*srr1*	PI-1b	PI-2b	–	–	+	+	+	+	+

Genes encoding hypervirulent adhesion (hvgA); fibrinogen binding protein (fbsB/A); laminin binding protein (lmb); hyaluronidase (hylB); C5a peptidase (scpB); ^f^ CAMP factor (cfa/cfb); serine-rich repeat protein encoding (srr). PIs, pilus islands; CCs, clonal complexes; STs, MLST sequence types.

The * cyl operon comprises 12 genes (cylX, cylD, cylG, acpC, cylZ, cylA, cylB, cylE, cylF, cylI, cylJ, and cylK); alp: gene encoding alpha-like protein variants (i.e., alphaC, alp1, alp2/3, and rib). (+, -) indicated presence and absence of genes.

### 3.5 Virulence factors

Most sequenced isolates (77.53%, n = 69/89) carried the gene encoding the surface protein Srr1, and only those belonging to CC17 (11.24%, n = 10/89) and ST1212 (3.37%, n = 3/89) harbored *srr2*, while seven isolates belonging to CC12 (n = 4) and CC19 (n = 3) lacked the gene (**Table 2**; **Supplementary Table 1**). The hypervirulent adhesion-encoding gene *hvgA* was detected as expected in all CC17 isolates (n = 10) (i.e., ST17 and ST2119) and surprisingly, in four other isolates belonging to ST1212 (n = 3) and ST934 (n = 1). Moreover, all isolates except five carried at least one of the four genes encoding surface proteins (Alpha C, Alp1, Alp 2/3, or Rib). Isolates lacking these genes belonged to ST1212 (n = 3) and ST26 (n = 2) (**Table 2**; **Supplementary Table 1**). Overall, the prevalence of these surface protein variants ranged from 17.98 to 29.21% and clear associations were observed between their presence and particular clonal complexes. For example, the majority of *alp2/3* (90%, n = 18/20) were detected in isolates from CC1. Otherwise, all isolates belonging to CC17 (n = 10) carried the *rib* gene, while all CC452 (n = 6) isolates and the majority of those from CC10/CC12 (n = 6/7) carried *alphaC.* In addition, all CC19 (n = 23) isolates had either *rib* or *alp1* (**Table 2**; **Supplementary Table 1**). On the other hand, the *cyl* operon genes (i.e., *cylX, cylD, cylG, acpC, cylZ, cylA, cylB, cylE, cylF, cylI, cylJ*, and *cylK*), which are involved in β-hemolysin and pigment production, were highly conserved among all sequenced isolates (100%, n = 89/89). In addition, the *scpB* (C5a peptidase)*, lmb* (laminin-binding protein), and *cfa/cfb* (pore-forming toxin) genes were also highly conserved, being detected in 100% (n = 89/89), 97.75% (n = 87/89), and 92.13% (n = 82/89) of the sequenced isolates, respectively. The hyaluronate lyase-encoding gene *hlyB* was detected in all isolates (88.76%, n= 79/89), except for 10 isolates belonging to CC19 expressing CPS III-1. In contrast, the gene encoding the fibrinogen-binding protein *fbsB* was only detected in 19 isolates belonging to CC23 (n = 3), CC17 (n = 10), and CC452 (n = 6), whereas *fbsA* was identified in a single isolate belonging to ST196 expressing serotype IV (**Table 2**).

### 3.6 Phylogenetic analysis

**Figure 2:**
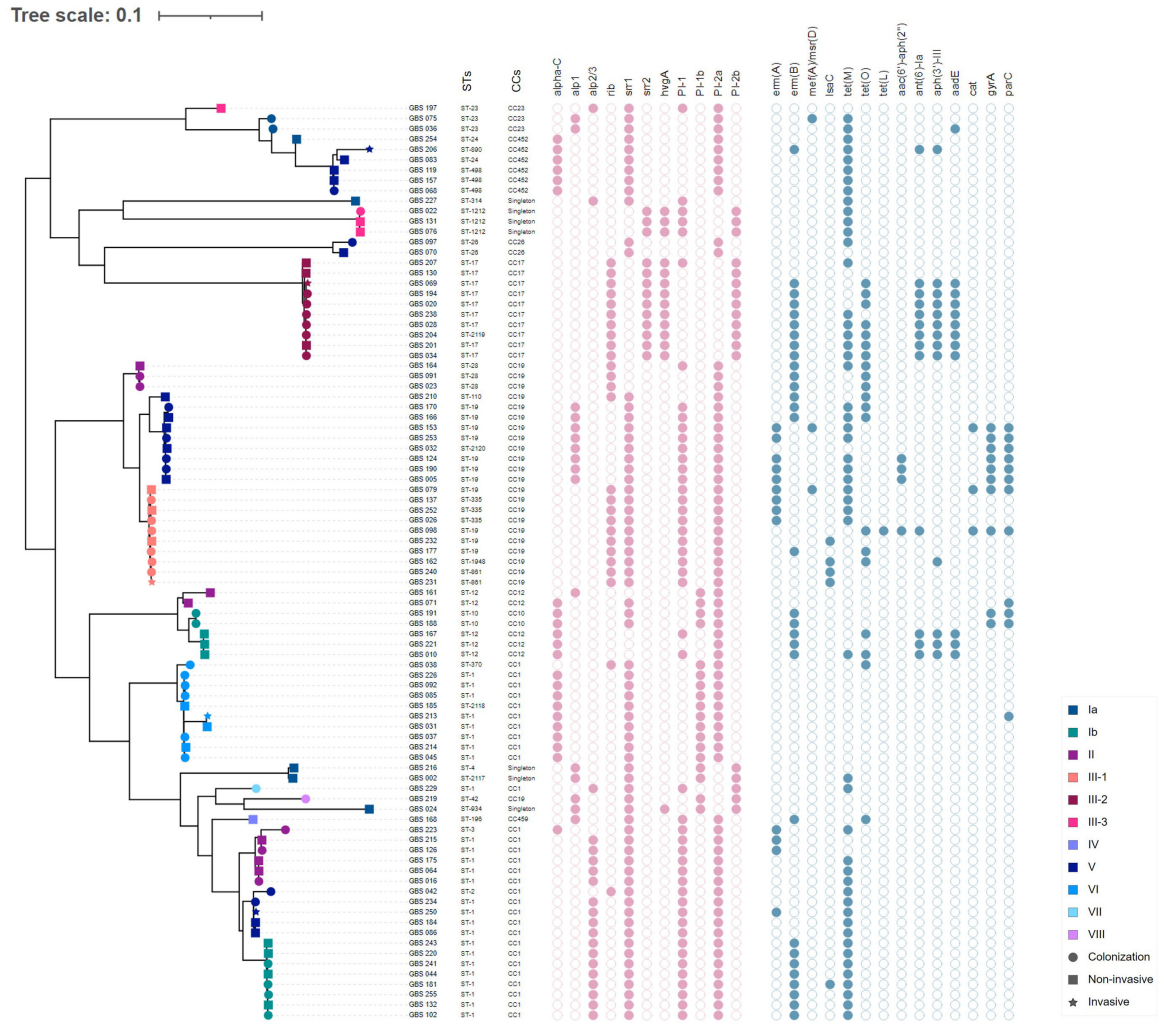
Whole-genome single nucleotide polymorphism (SNP)-derived mid-point rooted phylogenetic tree of 89 GBS isolates and their molecular characteristics. The phylogenetic tree was linked to the MLST type (first column) and the corresponding CC for each isolate (second column). The tip shapes show the infection type, whereas the color indicates the serotypes, as denoted by the legend bar on the bottom right. The filled and unfilled circles colored in pink indicate the presence or absence of the major surface protein genes (*alphaC, alp1, alp2/3, rib, srr1, srr2*, and *hvgA*) and pilus islands (PI-1, PI-2a, PI-1b, and PI-2b). Circles colored in blue indicate the presence or absence of acquired antimicrobial resistance genes or mutations in *gyrA* and *parC*.

**Figure 3:**
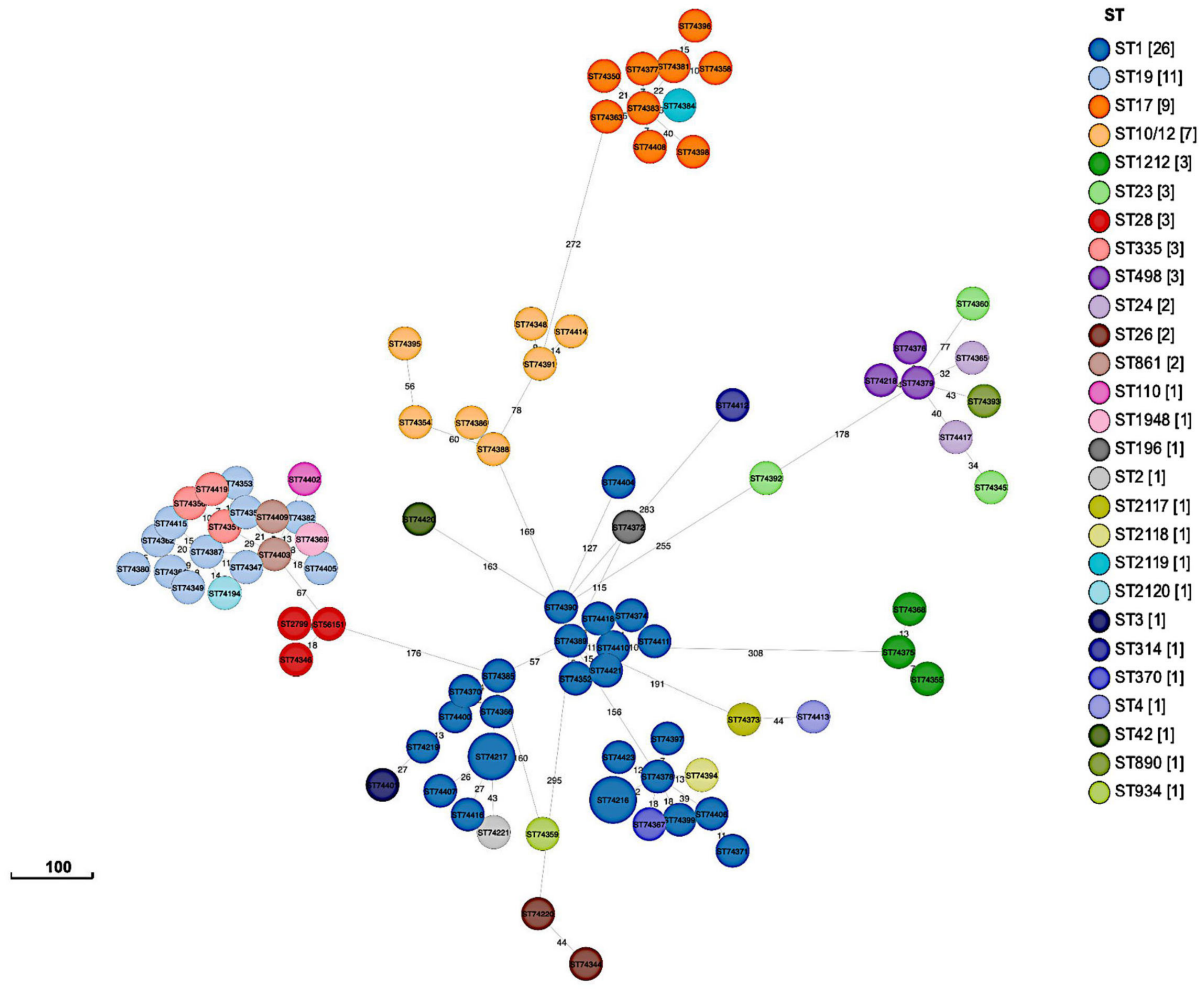
GrapeTree minimum-spanning tree showing cg-MLST of sequenced isolates (n = 89). The tree was constructed using the EnteroBase pipeline based on the *Streptococcus* scheme, comprising 1,918 target loci.

Phylogenetic analysis using a SNP-based approach grouped the isolates according to their CCs and STs (**Figure 2**). Overall, isolates within the same cluster tended to share similar combinations of virulence factors and PI variants, suggesting that these phylogroups represented distinct genetic lineages. Isolates belonging to the most common CC1 were further sub-divided into separate subgroups according to serotypes (i.e., Ib, II, V, VI, and VII); however, with no suggestions of any recent events of serotype switching. Indeed, all CC1 clusters exhibited the PI-1 and PI-2a loci and the surface protein Srr1, but harbored genes encoding different alpha-like proteins (Alpha C, Alp2/3, and Rib). Furthermore, CC19 clusters were dominated by the III/*rib*/*srr1*/PI-1+PI-2a (43.48%, 10/23) and V/*alp1*/*srr1*/PI-1+PI-2a (34.78%, 8/23) lineages, while most CC17 isolates (90%, 9/10) shared the same III/*rib*/*srr2*/P1-2b genetic background. The profiles of antimicrobial resistance determinants were found to be more diverse within the phylogenetic groups, suggesting that these genes might have been acquired through separate genetic events. The phylogeny of the sequenced isolates showed that those belonging to ST1212 and ST934, which also carried the CC17-specific adhesion *hvgA* gene, were genetically distinct, suggesting that they evolved and acquired the hypervirulence gene independently (**Figure 2**). Two pairs of sequenced isolates were found to be three and five SNPs apart from each other; they included isolates obtained from the same patients from different sites at different times. Relationships between all isolates were also reconstructed based on the GBS core-genome MLST (cg-MLST) scheme using the built-in pipeline of EnteroBase. Here also, cg-MLSTs separated the isolates by STs and their respective CCs (**Figure 3**; **Supplementary Table 1**). Overall, isolates belonging to separate CCs differed by up to 336 different loci, confirming high genetic diversity within the species (**Supplementary Table 2**). In particular, the isolates belonging to ST1212, ST934, and CC17, which all harbored the hypervirulent gene *hvgA*, clustered in separate groups and differed from each other by 306 to 324 loci (**Figure 3**; **Supplementary Table 1**). In addition, the two pairs of isolates from the same patients clustering together in the SNP-based approach belonged to the same cg-MLST types (i.e., cgST74217 and cgST74216) (**Figure 3**).

## 4 Discussion

The current study evaluated several important features of GBS isolates that are currently circulating in Saudi Arabia, including serotypes, sequence types, virulence factors, and molecular mechanisms of resistance to clinically important antibiotics, using whole-genome sequencing. Indeed, epidemiological studies characterizing the population structure of GBS isolates in the country and their resistance to antibiotics are important for the prevention and treatment of GBS infections and eventually for future vaccine implementation.

Sequence analysis inferred the serotypes of all isolates, including those that were non-typeable by standard PCR. Overall, the comparison of PCR-based serotyping with those inferred from whole-genome sequencing using the GBS-SBG database showed good concordance (i.e., 92.13%, n = 82/89), with only two isolates typed differently. In particular, whole-genome sequencing identified a handful of isolates belonging to serotypes VII and VIII, which were not previously reported in Saudi Arabia.

Sequenced isolates revealed 28 different STs; however, the majority of the detected STs belonged to four dominant human-associated lineages, including CC1, CC10/12, CC17, CC19, and the more recently described CC452, accounting together for 85.39% (n = 76/89) of all sequenced isolates (McGee et al., 2021; Rodgus et al., 2022). Of these, CC1 (33.71%, n = 30/89) and CC19 (25.84%, n = 23/89) were the most prevalent, followed to a lesser extent by the hypervirulent CC17 (11.24%, n = 10/89). Overall, the population structure of GBS isolates in this study correlates with previously published data reporting the same dominant CCs among colonizing and infecting isolates (Bianchi-Jassir et al., 2020; McGee et al., 2021; Rodgus et al., 2022). Nevertheless, the study showed that these main GBS lineages displayed varying levels of intra-lineage serotype diversity. Isolates from CC1, mainly ST1 (n = 26/30), were highly diverse and expressed five different serotypes (i.e., Ib, II, V, VI, and VII). Of the remaining, CC19, CC10/CC12, CC23, and CC452 also contained multiple STs and expressed multiple serotypes, which were also consistent with other published reports (McGee et al., 2021; Martins et al., 2022). The lowest diversity was observed among CC17 isolates (n = 10), which mainly belonged to ST17 (n = 9/10) and expressed serotype III-2 (**Figure 1**). Overall, the phylogeny of the studied collection did not show any evidence of recent serotype switching events, such as the previously described switch from serotype III to IV in CC17 or switch from serotype III to IV in CC17 or from serotype V to Ib within CC1 lineages (Lopes et al., 2018; McGee et al., 2021; Rodgus et al., 2022).

The sequenced isolates were checked for the presence of determinants associated with antibiotic resistance. Of particular concern was the high proportion of isolates with reduced susceptibility to macrolides and lincosamides, which are the antibiotics of choice for the treatment of GBS infections in patients with penicillin allergies. Here also, the molecular mechanisms of resistance to these antibiotics were consistent with those previously described for this species (Bergal et al., 2015; Jin et al., 2022; Rodgus et al., 2022; Khan et al., 2023). Phylogenetic analysis showed that resistance to macrolides and lincosamides was not necessarily linked to the expansion of certain CCs but was distributed across all detected CCs. This might explain the high rate of resistance to these antibiotics in the species that is likely to impact their use for the prevention and treatment of GBS infections. Isolates showing the cMLS_B_ phenotype were distributed across all major CCs and carried the methyltransferase-encoding gene *erm(B)*, while the isolates exhibiting the iMLS_B_ phenotype belonged to CC1 and CC19 and carried the *erm(A)* gene (Lopes et al., 2018; Martins et al., 2022; Rodgus et al., 2022; Khan et al., 2023). The few isolates showing the L phenotype coherently harbored the *lsa(C)* gene and belonged exclusively to CC19 expressing serotype III-1. On the other hand, the high rate of resistance to tetracycline was largely mediated by *tet(M)* and to a lesser extent by *tet(O)*, which was largely in agreement with previously published studies (Bergal et al., 2015; Jin et al., 2022; Rodgus et al., 2022; Khan et al., 2023). Sequence analysis linked gentamicin resistance with the presence of the gene encoding the bifunctional aminoglycoside-modifying enzyme AAC(6’)-APH(2’), and also identified other genes associated with resistance to kanamycin and streptomycin (i.e., *ant(6)-Ia*, *aph(3’)-III*, and *aadE*) in a small proportion of isolates (16.85%, n = 15/89), mainly belonging to CC17 and CC12. The coexistence of *erm(B)*, *tet(O)* with aminoglycoside resistance genes *ant(6)-Ia*, *aph(3’)-III*, and *aadE* in sequenced isolates was linked to the acquisition of the integrative conjugative element ICESag37, which was recently described in the species (Khan et al., 2023). Interestingly, multidrug resistance in a handful of isolates exhibiting resistance to macrolides, tetracycline, ciprofloxacin, and gentamicin was predominantly linked to ST19.

GBS pathogenicity is mediated by a set of virulence factors that may confer a selective advantage to the bacteria in terms of enhanced colonization, invasiveness, and virulence within the host cell. This study confirmed that PI-2b was exclusively associated with highly pathogenic CC17/III-2 isolates (Lu et al., 2018). In addition, all CC17 isolates expressed the hypervirulent adhesion HvgA, as expected. Surprisingly, the presence of this CC17-distinguishing gene was also detected in isolates belonging to ST1212 and ST934, which were phylogenetically distinct from CC17 isolates, suggesting that other highly virulent strains may be circulating within the species. Similarly, three *hvgA*-positive isolates of ST934 have been recently reported in Ethiopia and Egypt (Ali et al., 2020; Shabayek et al., 2023). Most sequenced isolates expressed Srr1 (77.53%, n=69/89), whereas the gene encoding the variant Srr2 was identified in CC17 isolates and, interestingly, in all ST1212 isolates. Srr2 has been reported to have a greater binding affinity for fibrinogen and plasminogen than Srr1, which further enhances the adherence to epithelial and endothelial cells in invasive niches and is therefore likely to contribute to the virulence of ST1212 isolates (Seo et al., 2013). In contrast to CC17, all ST1212 isolates expressed serotype III-3, had the P1-1 locus in addition to PI-2b, and lacked the *rib*-encoding genes. More than half of the isolates (62.92%, n = 56/89) carried the PI-1 and PI-2a variants and belonged to the main CC1, CC10/CC12, CC19, CC23, and CC459 (Martins et al., 2017; Del Carmen Palacios-Saucedo et al., 2022). In 2017, a study identified a novel PI-1 variant, named PI-1b, among isolates of serotypes Ia, Ib, II, III, VI, and VIII, although the significance of its presence remained unknown (Teatero et al., 2017). In our study, PI-1b was detected in combination with PI-2a in (n = 18) isolates of various serotypes (i.e., Ia, Ib, II, VI, and VIII), of which more than half (55.56%, n = 10/18) belonged to CC1 and expressed serotype VI.

Screening showed that genes for alpha family proteins were present in 94.38% (n = 84/89) of the isolates, suggesting that protein-based vaccines targeting this family of proteins would offer high protection. The Alpha C protein-encoding gene was present among various CCs (i.e., CC1, CC10, CC12, and CC452) and was commonly expressed on the surfaces of various serotypes, including Ib, V, and VI. In contrast, the gene encoding Alp2/3 was confined to CC1 isolates presenting serotypes Ib, II, V, and VII, whereas the gene encoding Alp1 was mainly present in CC19 isolates exhibiting serotypes V and VIII. In addition, one-third of the isolates carried the *rib* gene and mainly belonged to CC17 and CC19, presenting serotypes III-2 and III-1, respectively. However, isolates without any of the four alpha family members included those belonging to ST1212, which might potentially be highly virulent, and selective pressure of vaccine based upon the alpha-like surface proteins is likely to impact their prevalence. Other major virulence factors that mediate adhesion and invasion, including laminin-binding protein (*lmb*) and C5a peptidase (*scpB*) and those associated with the production of β-hemolysin/cytolysin (*cylE*), hyaluronidase (*hylB*), and adherence to CAMP factor pore-forming toxin (*cfb*), were nearly ubiquitous in all sequenced isolates (Udo et al., 2013). Interestingly, the majority of *fbsB*-encoding fibrinogen-binding proteins, thought to be important for GBS spread by promoting the invasion of host epithelial cells, were detected only in isolates belonging to CC17, CC23, and CC452 (Del Carmen Palacios-Saucedo et al., 2022). Finally, although in some cases, the serotype and surface protein genes were predictive of CC, there was no clear association between GBS infections and the presence of these genetic features. Indeed, the dominant CC1, CC17, and CC19 isolates were almost evenly distributed among colonized and infected GBS isolates.

## 5 Conclusions

Although the number of sequenced isolates was limited, the study provided for the first-time important insights into the genetic diversity of GBS isolates that are currently associated with human colonization and infections in the country. The data regarding the distribution of genetic lineages and the prevalence of genes associated with antibiotic resistance and virulence lay the foundation for future GBS surveillance studies in the country. The decrease in macrolide and lincosamide susceptibility and their distribution across all common human-associated clonal complexes is worrisome, corroborating the need for continued surveillance programs in our country to prevent the dissemination of GBS-causing diseases. In addition, the identification of the hypervirulent adhesion *hvgA* gene in non-CC17 isolates that were phylogenetically distinct, highlight the dynamic nature of this pathogen and underscores the need for more rigorous characterization of the genetic lineages causing infections.

## Data availability statement

The datasets presented in this study can be found in online repositories. The names of the repository/repositories and accession number(s) can be found below: https://www.ebi.ac.uk/ena/browser/view/PRJEB70279.

## Ethics statement

This study was conducted in accordance with ethical approval from the institutional review board (IRB Log Number: 22-172E) of the centralized committee of King Fahad Medical City. Written informed consent for participation was not required for this study, in accordance with national legislation and institutional requirements.

## Author contributions

MAlz: Conceptualization, Formal analysis, Investigation, Methodology, Resources, Supervision, Validation, Visualization, Writing – original draft, Writing – review & editing. MAlk: Conceptualization, Supervision, Writing – review & editing. AAly: Conceptualization, Resources, Writing – review & editing. MAld: Investigation, Writing – review & editing. AAla: Investigation, Writing – review & editing. GG: Investigation, Writing – review & editing. AS: Resources, Writing – review & editing. AA-H: Conceptualization, Resources, Writing – review & editing. MD: Conceptualization, Data curation, Formal analysis, Supervision, Validation, Visualization, Writing – original draft, Writing – review & editing.

## Supplementary material

Supplementary Table 2 CgMLST distance matrices for pairwise comparisons of the 89 GBS sequenced isolates.
