# Supplementary material for: Genomic insights into the diversity, virulence, and antimicrobial resistance of group B Streptococcus clinical isolates from Saudi Arabia
Source: Front Cell Infect Microbiol. 2024 Apr 22;14:1377993. doi: 10.3389/fcimb.2024.1377993 (PMC11070470; doi:10.3389/fcimb.2024.1377993)
Supplement: Supplementary file 3 [file DataSheet_1.docx]

Supplementary Material

**Genomic insights into the diversity, virulence, and antimicrobial resistance of Group B *Streptococcus* clinical isolates from Saudi Arabia**

Maha Alzayer^1,2,*^, Manal M. Alkhulaifi^1^, Ahmed Alyami^3^, Mohammed Aldosary^3^, Abdulaziz Alageel^3^, Ghada Garaween^2^, Atef Shibl^2^, Arif M. Al-Hamad^4^, Michel Doumith^5,*^

***Corresponding authors:**

Maha Alzayer

E-mail: maha.alzayer180@gmail.com, maalzayer@alfaisal.edu.

Michel Doumith

E-mail: [doumithm@gmail.com](mailto:doumithm@gmail.com)

# Supplementary Figures

## Supplementary Figures

**Supplementary Figure 1.** Distribution of clonal complexes among disease groups.

**Supplementary Figure 2.** Distribution of pilus island types by clonal complexes. PI-1 and PI-1b variants were distinguished by different colors.
